# Endoplasmic Reticulum Stress Is Involved in Glucocorticoid-Induced Apoptosis in PC12 Cells

**DOI:** 10.1155/2021/5565671

**Published:** 2021-02-12

**Authors:** Shanyong Yi, Weibo Shi, Min Zuo, Songjun Wang, Rufei Ma, Haitao Bi, Bin Cong, Yingmin Li

**Affiliations:** ^1^Hebei Key Laboratory of Forensic Medicine, Collaborative Innovation Center of Forensic Medical Molecular Identification, College of Forensic Medicine, Hebei Medical University, Shijiazhuang 050017, China; ^2^School of Forensic Medicine, Xinxiang Medical University, Xinxiang 453003, China

## Abstract

**Objective:**

The present study selected PC12 cells to construct a neuronal injury model induced by glucocorticoids (GC) in vitro, aiming to explore whether the endoplasmic reticulum stress (ERS) PKR-like endoplasmic reticulum kinase (PERK)-activating transcription factor 4 (ATF4)-C/EBP-homologous protein (CHOP) and inositol requirement 1 (IRE1)-apoptosis signal regulating kinase 1 (ASK1)-C-Jun amino-terminal kinase (JNK) signaling pathways are associated with the neuronal injury process induced by GC and provide morphological evidence.

**Methods:**

Cell models with different doses and different durations of GC exposure were established. The viability of PC12 cells was detected by the CCK-8 assay, and the apoptosis rate of PC12 cells was detected by the flow cytometry assay. The expression of microtubule-associated protein 2 (Map2); glucocorticoids receptor (GR); cellular oncogene fos (C-fos); and ERS-related proteins, glucose-regulated protein 78 (GRP78), p-PERK, p-IRE1, ATF4, ASK1, JNK, and CHOP, was observed by immunofluorescence staining.

**Results:**

The results of immunofluorescence staining showed that PC12 cells abundantly expressed Map2 and GR. The CCK-8 assay revealed that high-concentration GC exposure significantly inhibited the cell viability of PC12 cells. The flow cytometry assay indicated that high-concentration GC exposure significantly increased the apoptosis rate of PC12 cells. Immunofluorescence staining showed that GC exposure significantly increased the expression of C-fos, GRP78, p-PERK, p-IRE1, ATF4, ASK1, JNK, and CHOP. Treatment with ERS inhibitor 4-phenylbutyric acid (4-PBA) and GR inhibitor RU38486 attenuated related damage and downregulated the expression of the abovementioned proteins.

**Conclusion:**

High-concentration GC exposure can significantly inhibit the viability of PC12 cells and induce apoptosis. PERK-ATF4-CHOP and IRE1-ASK1-JNK pathways are involved in the above damage process.

## 1. Introduction

When stimulated by various internal and external factors, the HPA axis will be activated to increase the synthesis and secretion of GC, and proper secretion of GC can maintain body homeostasis [1]. Previous studies have shown that glucocorticoids play a vital role in the process of protecting cell lysosomal membranes, reducing toxin damage, anti-inflammatory, and antidamage [2, 3]. However, high levels of GC can downregulate the expression of GC receptors in tissue cells, which can inhibit the HPA axis, cause disorders of the hormone secretion system, inhibit the body's immune system, and reduce the body's resistance, eventually leading to metabolic disorders and severe damage [4]. Studies have shown that high concentrations of GC in the blood can cross the blood-brain barrier, causing neuronal injury and even death [5]. However, the specific mechanisms of the related damage remain unclear.

As an important component of the cell, the endoplasmic reticulum participates in a variety of physiological processes, such as protein synthesis, processing and trafficking, regulation of Ca^2+^ balance, and lipid synthesis [6]. Under normal circumstances, the protein folding ability of endoplasmic reticulum (ER) always matches with the body's protein synthesis ability [7]. When the body is stimulated by ischemia, hypoxia, injury, and other harmful elements, the microenvironment of ER will change, resulting in the accumulation of unfolded or misfolded proteins in the ER lumen, which can induce ERS [8]. Moderate ERS can be resolved by reducing protein translation and promoting the production of chaperone proteins; then, the ER can restore homeostasis [9, 10]. However, when ERS persists, PERK and IRE1 can dissociate from GRP78 and undergo autophosphorylation; then, the downstream ATF4-CHOP and ASK1-JNK signaling pathways are activated. Continuous high expression of CHOP and JNK can promote cell apoptosis [11–13].

However, it remains unclear whether the PERK-ATF4-CHOP and IRE1-ASK1-JNK signaling pathways are associated with the neuronal injury process induced by GC. Therefore, the present study selected PC12 cells to construct a neuronal injury model induced by GC in vitro, aiming to explore the mechanism of cell damage at the cellular level and provide morphological evidence.

## 2. Materials and Methods

### 2.1. Materials

The PC12 cell line was obtained from the American Type Culture Collection (Manassas, VA, USA). RPMI 1640 medium was purchased from Corning (Corning, NY, USA). Fetal bovine serum (FBS) was purchased from PAN Biotech (Aidenbach, Germany). Horse serum and nerve growth factor 2.5S (NGF 2.5S, 13257019) were purchased from Gibco (Grand Island, NY, USA). Sodium pyruvate solution and pen-strep solution were purchased from BI (Kibbutz Beit-Haemek, Israel). Dexamethasone (DEX), RU38486, and 4-phenylbutyric acid (4-PBA) were purchased from Sigma-Aldrich (St Louis, MO, USA). Cell freezing medium was purchased from ScienCell (Carlsbad, CA, USA). Cell Counting Kit-8 was purchased from MCE (Monmouth Junction, NJ, USA). The Annexin V-FITC/PI Apoptosis Detection Kit was purchased from BD Biosciences (San Diego, CA, USA). Antibodies against C-fos (ab222699), GRP78 (ab188878), ATF4 (ab186297), and CHOP (ab179823) were purchased from Abcam (Cambridge, MA, USA). Antibodies against p-PERK (PA5-37773) and p-IRE1*α* (PA1-16927) were purchased from Thermo Fisher (Waltham, MA, USA). Antibodies against ASK1 (A3271) and JNK (A11119) were purchased from ABclonal (Wuhan, HuBei, China). Antibodies against Map2 (GTX11267) and glucocorticoid receptor (GTX101120) were purchased from GeneTex (Alton Pkwy Irvine, CA, USA). Alexa Fluor™ 594 Donkey anti-rabbit IgG (H + L) (R37119) and Alexa Fluor™ 488 Donkey anti-mouse IgG (H + L) (A-21202) were purchased from Invitrogen (Carlsbad, CA, USA). All other chemicals and reagents used in the present study were analytical pure.

### 2.2. Cell Culture and Treatment

PC12 cells were cultured in RPMI 1640 medium supplemented with 7.5% fetal bovine serum, 5% horse serum, 100 U/mL penicillin/streptomycin, and 110 *μ*g/mL sodium pyruvate solution at 37°C in a humidified atmosphere of 5% CO_2_. PC12 cells were incubated with 50 ng/mL NGF 2.5S for 48 h to induce neurite formation and imitate neurons. After seeded in appropriate petri dishes, differentiated PC12 cell was used for subsequent experiments.

### 2.3. Cell Viability Assay

The viability of PC12 cells was detected by the CCK-8 assay. The differentiated PC12 cells were seeded into a 96 plate (4 × 10^4^ cells/well) and exposed to 25, 50, 100, 200, and 400 *μ*M DEX for 24 h or 100 *μ*M DEX for 24 h, 36 h, and 48 h. CCK-8 solution was added to each well at a dose of 10 *μ*L and incubated for 4 h at 37°C, then was detected at 450 nm by Multiskan Go (Waltham, MA, USA). Cell viability was defined and calculated by the following formula: [OD (experimental group) − OD (blank)]/[OD (Control group) − OD (blank)].

### 2.4. Flow Cytometric Analysis

The flow cytometric analysis experiment sets the following six groups: control, DEX (100 *μ*M), DEX + RU38486 (DEX: 100 *μ*M; RU38486: glucocorticoid receptor antagonist, 100 *μ*M), DEX+4-PBA (DEX: 100 *μ*M; 4-PBA: ERS inhibitor, 100 *μ*M), RU38486 (100 *μ*M), and 4-PBA (100 *μ*M).

After treated for 24 h, cells were collected and washed twice with PBS and then incubated with FITC-Annexin V and PI at room temperature for 10 min and protected from light, according to the manufacturer's instructions. The fluorescence of the cells was determined immediately by recording and analyzing 10,000 events using a flow cytometer (BECKMAN, S. Kraemer Boulevard Brea, CA, USA).

Live cells will show no staining by either the propidium iodide solution or Annexin V-FITC conjugate. Early apoptotic cells will be stained by the Annexin V-FITC conjugate alone. Late apoptotic cells will be stained by both the propidium iodide solution and Annexin V-FITC conjugate. Necrotic cells will be stained by the Propidium Iodide solution alone.

### 2.5. Immunofluorescence

Cells were fixed with 4% paraformaldehyde at room temperature for 20 min, then incubated by 0.2% Triton X-100 for 10 min on ice and followed by incubation in goat serum for 30 min. Next, the cells were incubated overnight at 4°C with antibodies specific for rabbit glucocorticoid receptor, GRP78, p-PERK, p-IRE1, ATF4, ASK1, JNK, CHOP, mouse C-fos, and Map2 and then incubated at 37°C with Alexa Fluor™ 594 Donkey anti-rabbit IgG (H + L) or Alexa Fluor™ 488 Donkey anti-mouse IgG (H + L). Finally, the cells counterstained with DAPI and images were acquired by a confocal laser scanning microscope (Leica, Ernst-Leitz-Strasse, Wetzlar, Germany).

In the present study, Image-Pro Plus 5.1 (Media Cybernetics, Houston, TX, USA) was used to count the number of cells and detect the fluorescence intensity (IOD) of all cells. The average IOD was used to illustrate the expression levels of the corresponding proteins.

### 2.6. Statistical Methods

The Kolmogorov–Smirnov test showed that the data were normally distributed in all groups (*P* > 0.1). The results are presented as mean ± SEM. Statistical analysis was performed by one-way ANOVA. The significance was defined as *P* < 0.05 for all statistical tests.

## 3. Results

### 3.1. Morphological and Structural Characteristics of Differentiated PC12 Cells

**Figure 1:**
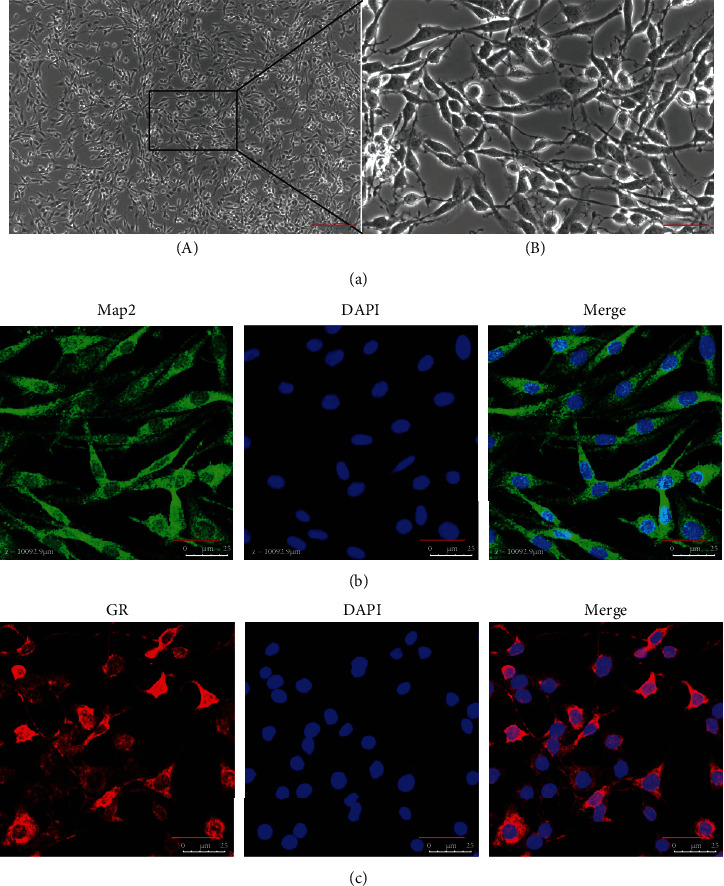
(a) The morphological characteristics of PC12 cells. (B) is the magnified area of (A). Bars = 200 *μ*m in (A) (magnification: ×100); bars = 50 *μ*m in (B) (magnification: ×400). (b, c) The expression of Map2 and GR in PC12 cells were detected by immunofluorescence. Bars = 25 *μ*m (magnification: ×800). Map2: microtubule-associated protein 2; GR: glucocorticoid receptor.

After treated with 50 ng/mL NGF for 48 h, PC12 cells presented polygonal or long fusiform and connected to each other through protrusions (Figure 1(a)).

The results of immunofluorescence experiments showed that differentiated PC12 cells expressed Map2 and GR abundantly (Figures 1(b) and 1(c)).

### 3.2. Cell Viability of PC12 Cells

**Figure 2:**
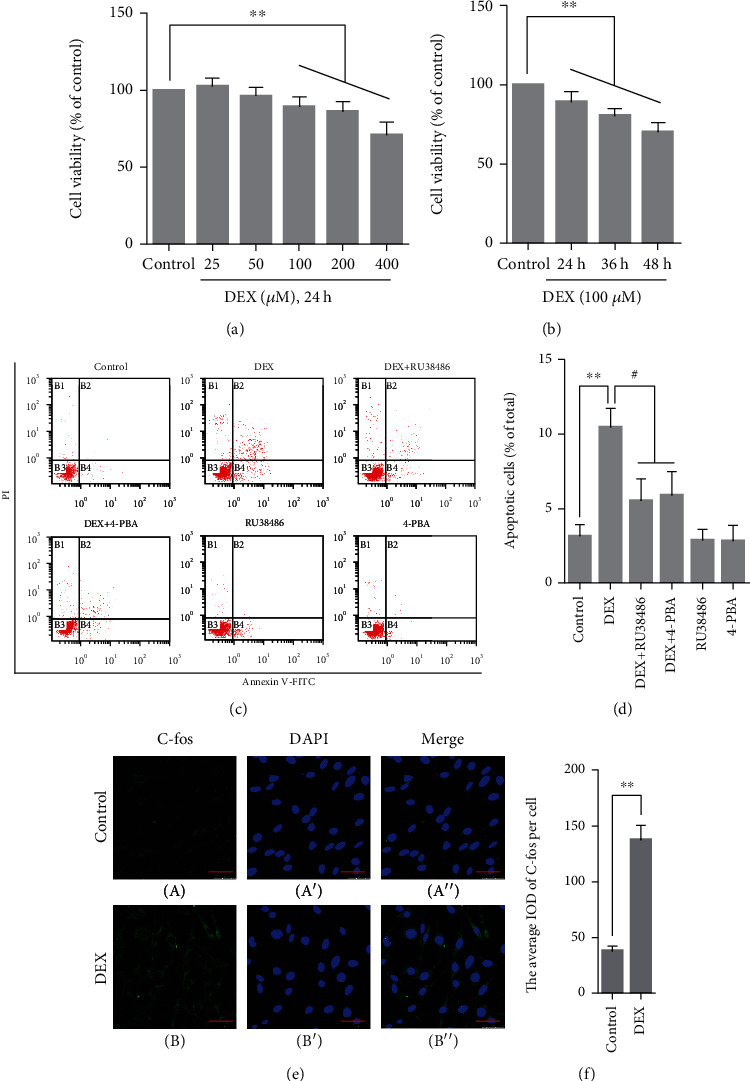
(a, b) The effect of DEX on the cell viability of PC12 cells. (c, d) The effect of DEX on the apoptosis rate of PC12 cells. (e) Representative images showing C-fos immunofluorescence in PC12 cells. (f) The average IOD of C-fos per cell. Bars = 25 *μ*m (magnification: ×800). The data are shown as the mean ± SEM, ^∗∗^*P* < 0.01 compared with the control group; ^#^*P* < 0.01 compared with the DEX group. DEX: dexamethasone; 4-PBA: 4-phenylbutyric acid; C-fos: cellular oncogene fos.

Compared with the control group (100.0 ± 0.0%), the viability of PC12 cells was significantly decreased with the raise of GC concentration (100 *μ*M (89.46 ± 2.10%, *P* < 0.01), 200 *μ*M (86.34 ± 2.18%, *P* < 0.01) ,and 400 *μ*M (70.98 ± 2.81%, *P* < 0.01)); compared with the control group, the viability of PC12 cells was significantly decreased after being treated with 100 *μ*M DEX for 24 h (89.46 ± 2.10%, *P* < 0.01), 36 h (80.52 ± 2.61%, *P* < 0.01), and 48 h (70.32 ± 3.41%, *P* < 0.01) (Figures 2(a) and 2(b)).

### 3.3. Apoptosis Rate of PC12 Cells

Compared with the control group (3.14 ± 0.44%), the apoptosis rate of PC12 cells remained at a low level in the RU38486 group (2.87 ± 0.41%, *P* > 0.05) and 4-PBA group (2.82 ± 0.60%, *P* > 0.05) and was significantly increased in the DEX group (10.46 ± 0.74%, *P* < 0.01); the apoptosis rate of PC12 cells was significantly decreased in the DEX + RU38486 group (5.52 ± 0.85%, *P* < 0.05) and DEX+4-PBA group (5.90 ± 0.91%, *P* < 0.05) (Figures 2(c) and 2(d)).

### 3.4. C-fos Protein Expression in PC12 Cells

Compared with the control group (37.9 ± 2.46), the C-fos expression increased significantly in the DEX group (137.5 ± 7.56, *P* < 0.01) (Figures 2(e) and 2(f)).

### 3.5. GRP78, P-PERK, P-IRE1, ATF4, ASK1, CHOP, and JNK Protein Expression in PC12 Cells

**Figure 3:**
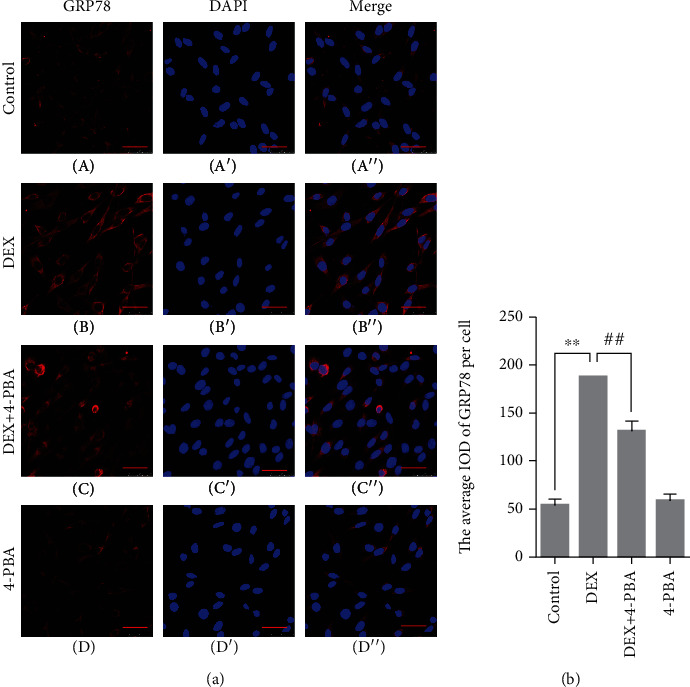
(a) Representative images showing GRP78 immunofluorescence in PC12 cells. (b) The average IOD of GRP78 per cell. Bars = 25 *μ*m (magnification: ×800). The data are shown as the mean ± SEM, ^∗∗^*P* < 0.01 compared with the control group; ^##^*P* < 0.01 compared with the DEX group. GRP78: glucose-regulated protein 78; DEX: dexamethasone; 4-PBA: 4-phenylbutyric acid.

Compared with the control group (54.0 ± 3.63), the GRP78 expression remained at a low level in the 4-PBA (58.9 ± 3.90, *P* > 0.05) group and was significantly upregulated in the DEX (188.0 ± 6.25, *P* < 0.01); compared with the DEX group, the GRP78 expression significantly decreased in the DEX+4-PBA (131.2 ± 5.97, *P* < 0.01) group (Figure 3).

**Figure 4:**
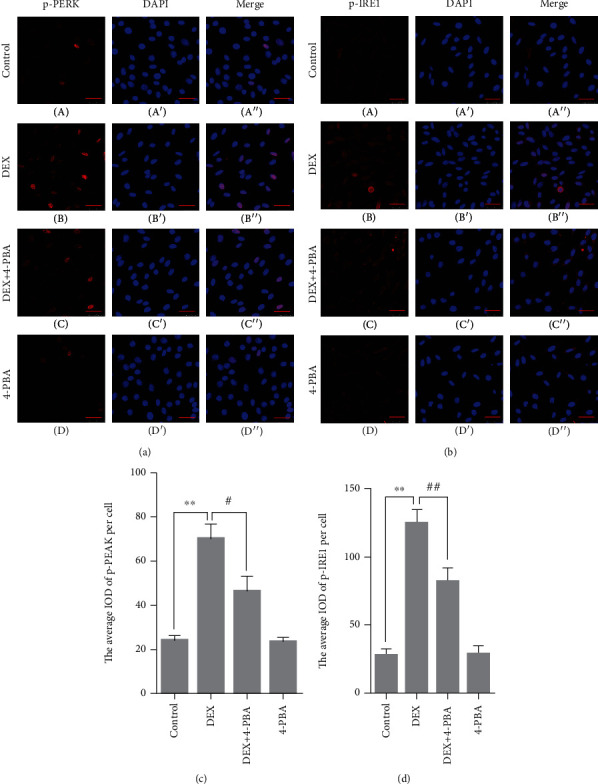
(a, b) Representative images showing p-PERK and p-IRE1 immunofluorescence in PC12 cells. (c, d) The average IOD of p-PERK and p-IRE1 per cell. Bars = 25 *μ*m (magnification: ×800). The data are shown as the mean ± SEM, ^∗∗^*P* < 0.01 compared with the control group; ^#^*P* < 0.01, ^##^*P* < 0.01 compared with the DEX group. PERK: PKR-like endoplasmic reticulum kinase; IRE1: inositol requirement 1; DEX: dexamethasone; 4-PBA: 4-phenylbutyric acid.

Compared with the control group (24.3 ± 1.21), the p-PERK expression remained at a low level in the 4-PBA (23.6 ± 1.27, *P* > 0.05) group and was significantly upregulated in the DEX (70.3 ± 3.70, *P* < 0.01); compared with the DEX group, the p-PERK expression significantly decreased in the DEX+4-PBA (46.4 ± 3.80, *P* < 0.05) group (Figures 4(a) and 4(c)).

Compared with the control group (27.9 ± 2.46), the p-IRE1 expression remained at a low level in the 4-PBA group (28.7 ± 3.51, *P* > 0.05) and was significantly upregulated in the DEX group (124.8 ± 5.74, *P* < 0.01); compared with the DEX group, the p-IRE1 expression significantly decreased in the DEX+4-PBA group (82.0 ± 5.79, *P* < 0.01) (Figures 4(b) and 4(d)).

**Figure 5:**
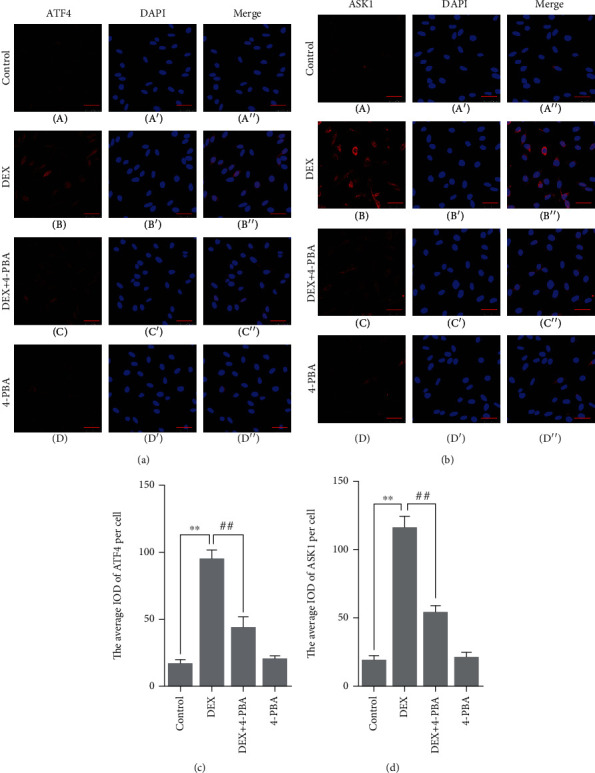
(a, b) Representative images showing ATF4 and ASK1 immunofluorescence in PC12 cells. (c, d) The average IOD of ATF4 and ASK1 per cell. Bars = 25 *μ*m (magnification: ×800). The data are shown as the mean ± SEM, ^∗∗^*P* < 0.01 compared with the control group; ^##^*P* < 0.01 compared with the DEX group. ATF4: activating transcription factor 4; ASK1: apoptosis signal regulating kinase 1; DEX: dexamethasone; 4-PBA: 4-phenylbutyric acid.

Compared with the control group (15.8 ± 1.95), the ATF4 expression remained at a low level in the 4-PBA (19.4 ± 1.58, *P* > 0.05) group and was significantly upregulated in the DEX (94.0 ± 4.16, *P* < 0.01); compared with the DEX group, the ATF4 expression significantly decreased in the DEX+4-PBA (42.8 ± 4.96, *P* < 0.01) group (Figures 5(a) and 5(c)).

Compared with the control group (18.0 ± 2.28), the ASK1 expression remained at a low level in the 4-PBA (20.1 ± 2.37, *P* > 0.05) group and was significantly upregulated in the DEX (115.0 ± 5.14, *P* < 0.01); compared with the DEX group, the ASK1 expression significantly decreased in the DEX+4-PBA (53.1 ± 3.17, *P* < 0.01) group (Figures 5(b) and 5(d)).

**Figure 6:**
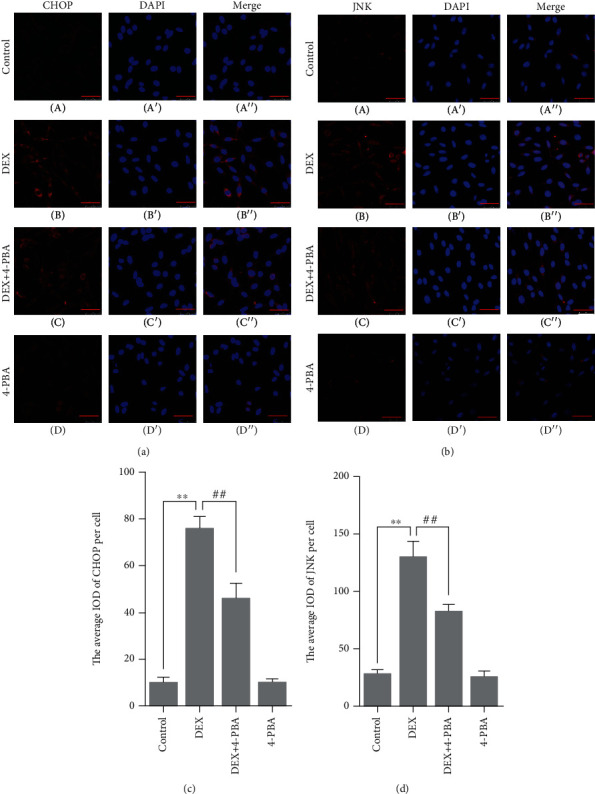
(a, b) Representative images showing CHOP and JNK immunofluorescence in PC12 cells. (c, d) The average IOD of CHOP and JNK per cell. Bars = 25 *μ*m (magnification: ×800). The data are shown as the mean ± SEM, ^∗∗^*P* < 0.01 compared with the control group; ^##^*P* < 0.01 compared with the DEX group. CHOP: C/EBP-homologous protein; JNK: C-Jun amino-terminal kinase; DEX: dexamethasone; 4-PBA: 4-phenylbutyric acid.

Compared with the control group (9.42 ± 1.55), the CHOP expression remained at a low level in the 4-PBA (9.41 ± 1.16, *P* > 0.05) group and was significantly upregulated in the DEX (75.34 ± 3.22, *P* < 0.01); compared with the DEX group, the CHOP expression significantly decreased in the DEX+4-PBA (45.38 ± 4.09, *P* < 0.01) group (Figures 6(a) and 6(c)).

Compared with the control group (26.78 ± 2.33), the JNK expression remained at a low level in the 4-PBA (24.3 ± 3.23, *P* > 0.05) group and was significantly upregulated in the DEX (128.6 ± 8.46, *P* < 0.01); compared with the DEX group, the JNK expression significantly decreased in the DEX+4-PBA (81.27 ± 4.27, *P* < 0.01) group (Figures 6(b) and 6(d)).

## 4. Discussion

PC12 is a cell line derived from rat adrenal pheochromocytoma. After culturing in the culture medium containing neurotrophic factors, the morphology and physiological functions of PC12 cells can change and differentiate into sympathetic nerve cells. The differentiated PC12 cell has general characteristics of neuroendocrine cells; so, it is widely used in neurophysiology and neuropharmacology research [14, 15]. In the present study, the differentiated PC12 cells expressed Map2 abundantly, which indicated that the differentiated PC12 cell has the basic structural characteristics of neurons. GC exerts biological effects by recognizing and binding the glucocorticoid receptors (GC receptor, GR). GR includes two subtypes, termed *α* and *β*, and glucocorticoids mainly exert their physiological and pharmacological functions by binding to the *α* receptors. *α* receptors are expressed in almost all tissues and nucleated cells, and the content in most cells far exceeds *β* receptors [16]. The results of the present study showed that PC12 cells expressed GR*α* receptors abundantly. The above experimental results suggested that differentiated PC12 cells are suitable for the study of the mechanism of GC-induced neuronal injury.

When stimulated by moderate internal and external factors, organisms can maintain body homeostasis by appropriately increasing GC synthesis and secretion. However, excessive stress can induce mass secretion of GC. High-concentration GC can penetrate the blood-brain barrier and cause neuronal injury or even death, which can lead to a series of mental disorders [17, 18]. In the present study, the results of CCK-8 experiments showed that high-concentration GC exposure can significantly inhibit cell viability and reduce the cell survival rate; the results of flow cytometry experiments showed that high-concentration GC treatment significantly increased the apoptosis rate of PC12 cells, and the application of GR antagonist RU38486 significantly reduced the apoptosis rate of PC12 cells. C-fos is an immediate early gene. The transcription of C-fos can be promoted rapidly by internal and external stimuli [19]. Under normal conditions, the expression level of C-fos is extremely low. Therefore, the upregulation of the C-fos expression can be regarded as a sign that neurons are subjected to damaging stimulation [20]. In the present study, after treated with 100 *μ*M DEX for 2 h, the expression of C-fos in PC12 cells significantly increased, indicating that cells can be rapidly activated by GC. The above results showed that high concentrations GC can lead to cell damage and even apoptosis, suggesting that the GC-induced neuronal injury model has been successfully established.

Previous studies have shown that GC may induce apoptosis by activating mitochondria- and endoplasmic reticulum-dependent pathways; therefore, the detailed mechanism requires further investigation. ERS is an adaptive response to internal and external stimuli formed during the process of complex biological evolution. Moderate ERS can upregulate the expression of the molecular chaperone GRP78, which can enhance the body's ability to deal with unfolded or misfolded proteins and reduce protein synthesis, then restore cell homeostasis and function [21]. However, when ERS persists, PERK and IRE1 can be activated [22]. Activated PERK can form oligomers through autophosphorylation and initiate the downstream ATF4-CHOP pathway [23, 24]. IRE1 has two isoforms that exist in different parts. IRE1*α* is generally distributed in mammalian cells, and IRE1*β* is expressed only in intestinal epithelial cells [25]. Similar to PERK, excessive ERS can lead to the autophosphorylation of IRE1, which then initiate the downstream ASK1-JNK pathway by its phosphokinase activity. As apoptotic factors, the continued expression of JNK and CHOP can cause body injury by blocking the cell cycle and inducing apoptosis [26–29]. In the present study, after treated with 100 *μ*M DEX for 24 h, the results showed that the treatment of high-concentration GC leaded to the increased expression of ERS-related proteins GRP78, ATF4, CHOP, ASK1, and JNK in PC12 cells and increased phosphorylation levels of PERK and IRE1. While the application of ERS inhibitor 4-PBA significantly reduced the phosphorylation and expression levels of the abovementioned proteins and decreased the apoptosis rate of PC12 cells, the PERK-ATF4-CHOP and IRE1-ASK1-JNK pathways were involved in the GC-induced apoptosis process of PC12 cells.

In conclusion, the present study clearly demonstrated that high-concentration GC exposure can significantly inhibit the viability of PC12 cells and induce apoptosis. PERK-ATF4-CHOP and IRE1-ASK1-JNK pathways are involved in the above damage process. We believe these novel findings will provide a pathomorphological foundation for the mechanism of GC-induced neuronal injury.

## Data Availability

The data used to support the findings of this study are included within the article.

## Supplementary Materials

Supplementary Materials The supplementary file includes the graphical abstract.
