# Supplementary material for: Endoplasmic Reticulum Stress Is Involved in Glucocorticoid-Induced Apoptosis in PC12 Cells
Source: Anal Cell Pathol (Amst). 2021 Feb 12;2021:5565671. doi: 10.1155/2021/5565671 (PMC7895572; doi:10.1155/2021/5565671)
Supplement: Supplementary Materials — The supplementary file includes the graphical abstract. [file 5565671.f1.docx]

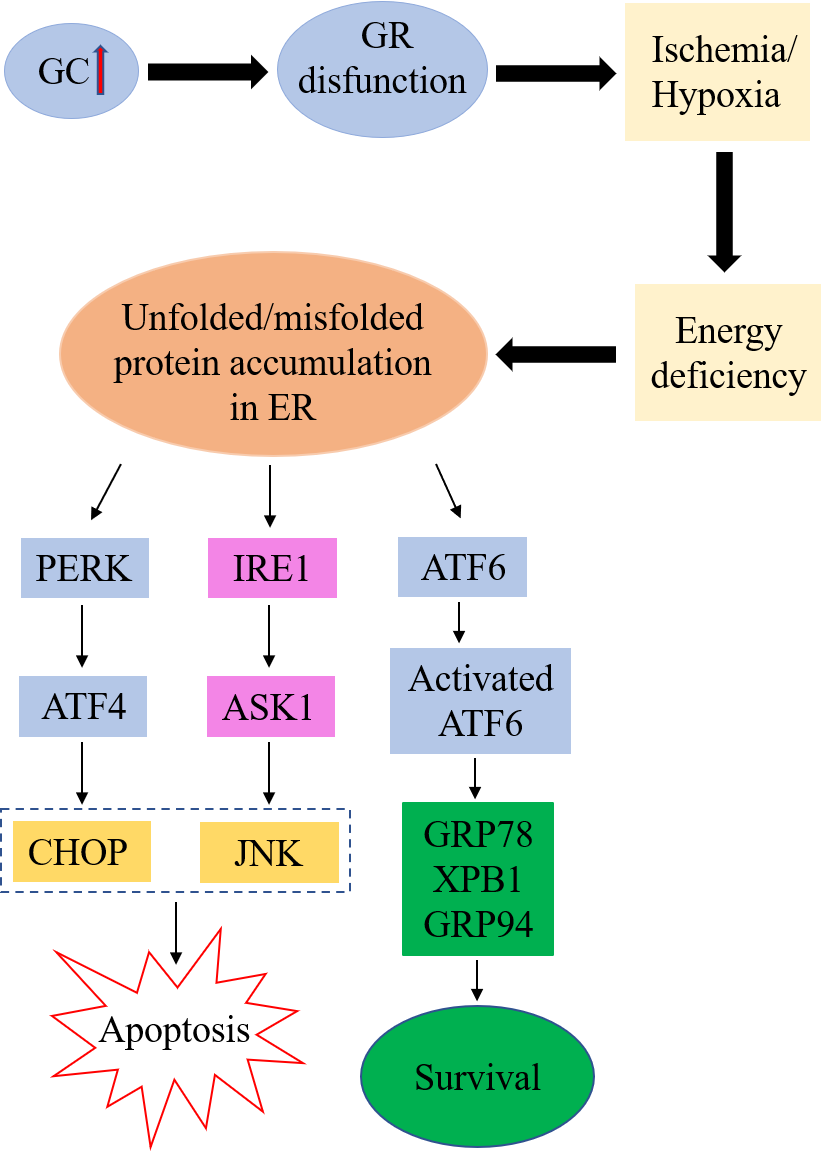


GC: Glucocorticoid

GR: Glucocorticoid receptor

ER: Endoplasmic reticulum

PERK: PKR-like endoplasmic reticulum kinase

IRE1: Inositol requirement 1

ATF4: Artificial transcription factor 6

ASK1: Recombinant apoptosis signal regulating kinase 1

JNK: C-Jun amino-terminal kinase

CHOP: C/EBP homologous protein

GRP78: Glucose-regulated protein 78

XPB1: Xeroderma pigmentosum complementation group B 1

GRP94: Glucose-regulated protein 94

ATF6: Activating Transcription Factor 6
